# The London money market and non-British bank lending during the first globalisation: evidence from Brazil

**DOI:** 10.1007/s11698-024-00284-5

**Published:** 2024-04-15

**Authors:** Wilfried Kisling, Marco Molteni

**Affiliations:** 1 Faculty of History, University of Oxford, Oxford, UK; 2 WU Wirtschaftsuniversität Wien, Vienna, Austria; 3 Geneva Graduate Institute, Geneva, Switzerland

**Keywords:** London money market, First wave of globalisation, Non-British overseas banks, German foreign banks, Sterling dominance, International banking before 1914, N23, F34, G15, N26, G21, E44

## Abstract

This study examines the relationship between the London money market (LMM) and the credit provision of non-British overseas banks in peripheral economies during the first wave of globalisation. Using monthly data between 1889 and 1913, we find a positive relationship between the amount of credit authorised by the German *Brasilianische Bank für Deutschland* in Brazil and the spread between the London market and floating rate. Our results suggest that increased demand for foreign bills and/or decreased borrowing costs in the LMM leads to an increase in credit supply. We use the impact of annual tax payments on the spread between the market and floating rate as an instrumental variable (IV) to show that this relationship is causal. Although there is a significant amount of literature on London’s historic role as a global financial centre and a growing number of studies on foreign banking history, little quantitative evidence is available about the connection between the two. This study bridges this gap.

## Introduction

In the nineteenth century, London was the world’s financial centre, and a country’s ability to finance its trade and government was highly dependent on its access to the London money market (LMM) (Kindelberger 1974; Flandreau and Jobst 2005; Accominotti et al. 2021). However, as the global capital and trade markets became more interconnected and competitive in the late nineteenth century, some nations began questioning their financial reliance on London and looking for alternatives. One strategy was to establish a foreign banking presence, which had the capacity to provide informational and financial support to their businesses and commerce abroad and to provide alternatives to sterling as key trade currency. While research has demonstrated the benefits of foreign banks in supporting a nation’s trade and business overseas (Kisling 2020, 2023), attempts to break the dominance of sterling and the LMM were less successful (Tilly 1992; Schneider 2019).

This paper empirically examines the relationship between the LMM and the credit provision of non-British overseas banks in peripheral economies during the first wave of globalisation. Specifically, it studies whether fluctuations in the LMM influenced the credit supply of the German foreign bank *Brasilianische Bank für Deutschland* from its establishment in Brazil in 1889 until the outbreak of WWI. While the idea that the LMM affected the credit provision of non-British banks might not be novel, to the best of our knowledge, this is the first time this relationship is tested empirically and documented. We also show that this is causal using an instrumental variable (IV) approach that relies on the historical operation of the LMM. While the literature focuses chiefly on the market discount rate, we focus our attention on the spread between this rate and the floating rate, i.e. the rate at which London banks lent money overnight. We argue that the spread between the two is key for foreign banks that did not have a direct presence in London, and had to rely on bill brokers and discount houses through their London correspondents.

In general, a larger spread signals that the conditions of the LMM are not stringent, i.e. the market is liquid and ample funds can be employed to rediscount bills. More in details, two principal mechanisms determined the link between the spread of the London market discount rate on prime bills (market rate) and the day-to-day loans rate (floating rate) and the credit supplied by foreign banks, which primarily used sterling-denominated bills of exchange to finance international transactions. The market rate reflects the price at which bills are bought and sold on the discount market, while the floating rate reflects the cost of short-term borrowed capital made available by London banks to banks and other agents, such as bill brokers and discount houses. The first mechanism involves London joint-stock banks. When the market rate is higher, it becomes more profitable for London banks to discount foreign bills. At the same time, low floating rates indicate that London banks have ample availability of funds to invest. As a result, demand for foreign bills in London increases when the spread between the market and floating rates is larger. The second mechanism involves bill brokers and discount houses, which play a key role in the discount market by intermediating between acceptors and final investors in bills of exchange by buying bills from the former and selling them to the latter^1^. These actors rely on narrow margins between the price of buying and selling bills for their profits, and therefore require large volumes of transactions to be profitable. Yet, their own capital is limited and most of their funds are borrowed from London bankers at the floating rate. A larger spread between the market and floating rate means that bills and borrowed money are relatively cheaper, allowing bill brokers and discount houses to intermediate larger amounts of bills. In some cases, discount houses do not re-sell the bills, but instead hold them until maturity. In this case, a larger spread between the market and floating rates means that they can borrow cheaply and lend at high-interest rates.

The case of the *Brasilianische* is significant for several reasons. Firstly, it represents the importance of foreign banks in the internationalisation of Germany, a rapidly emerging economy at the time. By the turn of the century, it had become the second most important trade nation behind the UK and the third largest economy in the world (Daudin et al. 2010; Carreras and Josephson 2010)^2^. German foreign banks were key to this successful expansion by providing financial services and informational assistance abroad (Hertner 2012). The *Brasilianische* is commonly acknowledged by coeval observers as a successful and representative blueprint of German overseas banking during the first globalisation (Diouritch 1909; Hurley 1914). Secondly, the emerging economies of Latin America were a major destination for European foreign banking during this period, with foreign banks playing an essential role in the region’s economic development and integration into international trade markets^3^. According to Jones (1993), Latin America was one of the markets where, after their first-mover advantage, British multinational banks faced harsher competition, particularly from German banks. Finally, the case of the *Brasilianische* highlights the competition faced by British banks from non-British banks, while also demonstrating London’s continued centrality in the global financial network. Despite attempts by German foreign banks to promote the independence of German international commerce from London and to offer the German mark as an alternative international currency, we find that they could not break away from the hegemony of the pound sterling.

The *Brasilianische Bank für Deutschland*, founded in 1887 in Hamburg, aimed to facilitate trade relations between Germany and Brazil. It opened its first branch in Rio de Janeiro in 1889, providing direct credit and primarily using bills of exchange as a financing instrument. However, by statute, the bank was not allowed to use funds denominated in the Brazilian currency, the Milreis, for international business. To avoid exchange rate risks, it drew on European places that offered the most favourable conditions. As a result, over 80% of the bills of exchange it discounted were denominated in pounds sterling. Despite not having a branch in London, the bank had direct access to the LMM through its London agents and correspondent banks, and later through the London subsidiary of its mother institution, the *Disconto-Gesellschaft*.

Using an OLS regression, we find a positive relationship between the monthly amount of credit lines authorised by the bank and the spread between the London market and floating rate. Our results suggest that the amount of credit authorised by the *Brasilianische* bank increases when there is (i) increasing demand for foreign bills in the London market relative, (ii) a decrease in borrowing costs for bill brokers and discount houses in London.

Our findings are not affected by reverse causality between our dependent and independent variables, as it is unlikely that the credit provision of the *Brasilianische* would impact London’s interest rates. However, our model may be subject to omitted variable bias. We include time-fixed effects and control for several additional variables to address this issue. We also employ an instrumental variable (IV) strategy to test the robustness of our results. Specifically, we use annual tax revenue collection in Great Britain and its effect on the spread of the market and floating rate as our IV. Individuals and companies based in Great Britain had to pay their annual income and other taxes at the end of March. Consequently, throughout the months of February and March, large amounts of money deposited at British joint-stock banks were withdrawn and transferred to the Government accounts at the Bank of England. This contraction in funds forced the joint-stock banks to reduce the amount of money they had available for daily loans. This led to an increase in the floating rate, and hence the spread decreased. As the *Brasilianische* Bank was not present in Britain and not impacted by British fiscal dynamics, we consider this shock to be exogenous. Our IV estimations support the findings of our OLS regression.

The remainder of this paper is organised as follows. The next section gives the historical context describing the Brazilian economy in the late 19th and early twentieth century, the history of the *Brasilianische Bank*, and the operation of the LMM. Section 3 presents our empirical strategy focusing on our main model. Section 4 discusses our identification strategy and confirms our primary model’s results. The final section concludes the paper.

## The *Brasilianische* Bank in the Brazilian Economy

Exports, primarily coffee and rubber, drove the Brazilian economy in the late 19th and early twentieth centuries^4^. Between 1889 and 1919, coffee comprised over 57% of Brazilian exports and 71% of the world’s total production. Rubber’s share in exports grew from 14.2 to 25.6%. The coffee industry faced a major setback due to overproduction and saturation in the international market, leading to a price drop. This drop decreased production between 1900 and 1905, until the Brazilian government stepped in as a direct buyer in 1905/6. Rubber exports also declined when Southeast Asian producers entered the market in the 1910s, accounting for only 5% of Brazilian exports in 1919 (Strasser 1924, Abreu and Bevilaqua 1996; Bértola and Ocampo 2010, see also Absell and Tena-Junguito 2016; and Klasing, & Milionis 2014). Table 1 shows the export shares of the principal commodities of Brazil between 1870 and 1919. Table 2 shows the main trading partners of Brazil in 1910. Germany ranked third in terms of Brazilian exports and second in Brazilian imports.

**Table 1:** Commodity export shares Brazil (% of total), 1870–1919. *Source* Abreu and Bevilaqua 1996 p. 9

Year	Coffee	Sugar	Cotton	Rubber	Total
1870–79	56.3	11.8	9.7	5.5	83.3
1880–89	60.5	10.6	4.4	7.6	83.1
1890–99	65.4	6.1	2.5	14.2	88.2
1900–09	53.1	1.5	2.3	25.6	82.6
1910–19	52.1	2.4	1.7	16.4	72.6

**Table 2:** Brazil’s main trading partners (1910). *Source* Dedinger and Girard (2017)

Exports		Imports	
USA	22,855,681	United Kingdom	13,676,221
United Kingdom	14,579,528	Germany	7,607,898
Germany	7,465,804	USA	6,127,582
France	5,309,449	France	4,539,270
Netherlands	3,241,507	Argentina	4,071,564

Trade flow in £ Pounds

These exports obviously needed to be financed, and the principal foreign actors in the Brazilian banking business were the British and the Germans (Haber 1997; Briones and Villela 2006).^5^ The driving force of British banking engagement in the second half of the nineteenth century in Brazil was the increasing investment possibilities in the capital markets and infrastructure projects (Hurley 1914; Triner 2006). The integration of the Brazilian economy into global markets made large-scale infrastructure work necessary. Mainly driven by coffee, the export boom triggered the construction of harbours and, most importantly, railway systems. The two largest and most influential German and British financial institutions in 19th-century Brazil were the *Brasilianische Bank für Deutschland* and the *London and Brazilian Bank*. The latter was the first foreign bank established in Brazil, opening its first branch in 1863 in Rio de Janeiro (Orbell and Turton 2001; Young 1991). By the beginning of the XX century, the *British Bank of South America* (firstly established in Brazil as the Brazilian and Portuguese Bank) had overtaken the *London and Brazilian* as the largest foreign bank in terms of deposits and credit (Table 3). The *Brasilianische Bank für Deutschland* was the second most important foreign bank.^6^

**Table 3:** Deposits and lending of main banks operating in Rio de Janeiro (Jan 1911). *Source* Retrospecto Commercial do Jornal do Commercio (1912)

Bank	Discounts and current accounts	Deposits and creditor accounts
Banco do Brasil	47,783,105	131,722,237
British Bank of South America	24,939,012	35,289,217
Brasilianische Bank fur Deutschland	23,735,277	22,454,431
Banco Commercial	12,087,988	13,583,182
Banco Mercantil do Rio de Janetro	8,162,655	3,406,308
London & River Plate Bank	6,708,650	12,706,099
Banco do Commercio	6,188,239	4,515,341
London & Brasillian Bank	5,414,998	15,985,422
Banco da Lavoura e do Commercio	3,735,859	1,990,006
Banco Espanol del Rio de la Plata	1,755,615	1,239,427

Figures in Milreis

In 1887, the *Disconto-Gesellschaft* in Berlin and the *Norddeutsche Bank* in Hamburg founded the *Brasilianische Bank für Deutschland*. It opened its first branch in 1889 in Rio de Janeiro. Both banks, independently of each other, had already shown an interest in expanding to Latin American markets in previous years. While the *Disconto* was interested in entering Brazil’s infrastructure and railway construction business, the *Norddeutsche* had already been an important player in Brazil’s export and import sectors in previous decades (Brasilianische Bank für Deutschland 1912). Yet, the risk and capital intensity had prevented an earlier market entry and ultimately led to the decision of *Norddeutsche* and *Discontobank* to combine their efforts in establishing a foreign bank. The focus of German banks on financing trade becomes even more evident when compared to their British counterparts. A direct comparison of the amounts of bills discounted by the *Brasilianische Bank für Deutschland* and the London & Brazilian Bank reveals that the German bank financed more bills (see Figs. 10, 11 in the Appendix).

The *Brasilianische* Bank was the only German bank operating in Brazil until 1911, when the *Deutsche Überseeische* and the *Deutsch-Südamerikanische* Bank were established in Rio de Janeiro. In 1913, together these three banks possessed over nine branches in Brazil. The *Brasilianische,* however, was the only institution that exclusively concentrated its business on the Brazilian market. In the same year, three British banks with twenty-two branches were operating in Brazil; the *London and Brazilian*, the *London and River Plate*, and the *British Bank of South America* (see Hurley 1914; Hauser 1901). Yet, the increasing competition from German banks had its impact. In 1906, British banks held some 77% of the foreign deposits in the major financial centres: In 1930, this figure was down to 31%. German banks ‘were by far the second most relevant actors in the region. (…) In terms of indicators such as total deposits, paid-in capital or profits, they were far bigger than their continental competitors, such as the French’ (Briones and Villela 2006, p.5–6).

The *Brasilianische* was closely linked to its mother institutions and the European money market. In 1887, the joint-stock capital of the *Brasilianische* was 2.5 Million mark, of which 1.5 Million were deposited at the *Disconto-Gesellschaft* and 1 Million at the *Norddeutsche Bank* (Supervisory Report Brasilianische, March 1888). Furthermore, both mother institutions held most of *Brasilianische’s* (Brasilianische Bank für Deutschland 1912) stock shares. The bank’s headquarters, including the directorate and the supervisory board, were in Hamburg, Germany^7^, and every credit line granted by the *Brasilianische* had to be confirmed by the supervisory (Supervisory Reports Brasilianische).

**Fig. 1:**
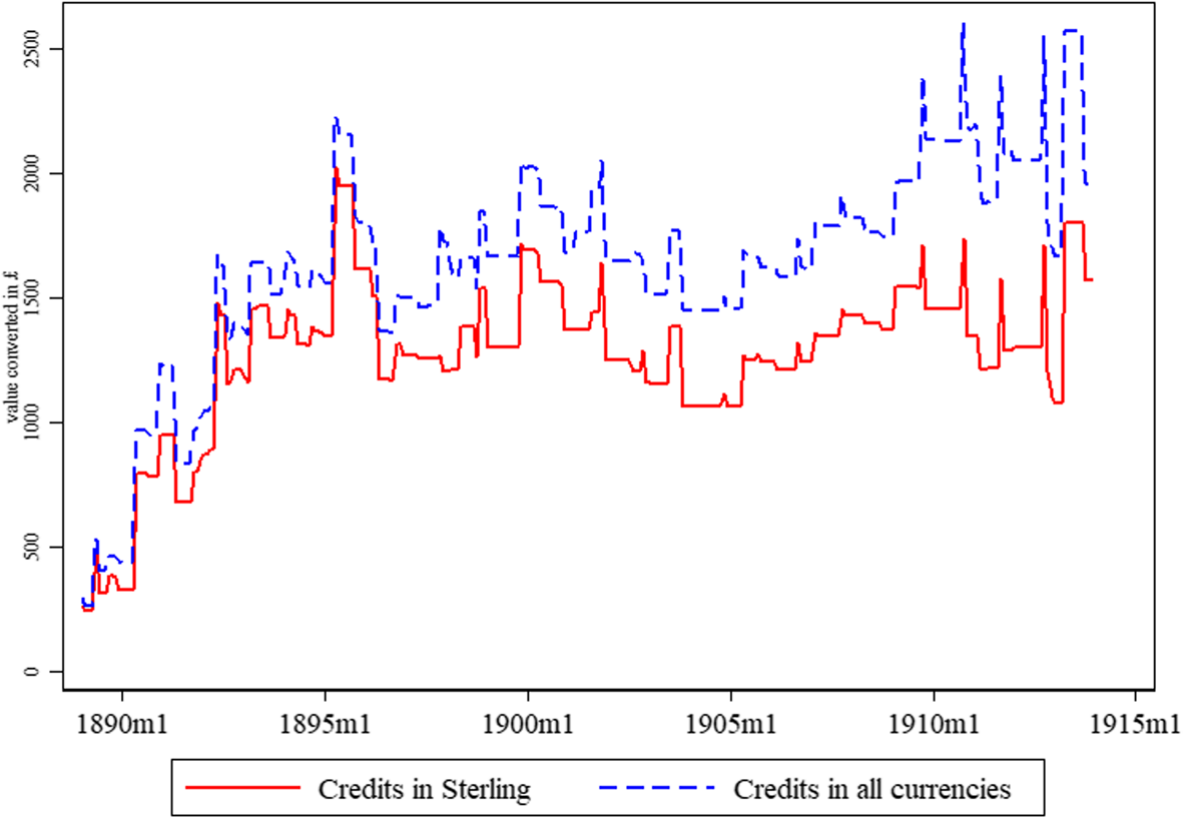
Monthly authorised credit lines of the *Brasilianische* Bank—bills discounting and direct credit—all currencies and in sterling pounds, 1889–1913

The *Brasilianische* financed business in Europe and in Brazil in multiple currencies.^8^ Yet, its use of domestic capital denoted in Milreis was restricted to finance business in Brazil only. This was the bank’s attempt to protect itself against the high volatility of the Brazilian currency and the resulting exchange rate risks (Brasilianische Bank für Deutschland 1912; Diouritch 1909). The capital to finance international business had to be acquired exclusively by drawing on Hamburg and Berlin, and, if more favourable conditions were available, on the international financial centres in foreign currencies (Supervisory Report Brasilianische, March 1888). From the 1870s, Germany tried to establish the German Mark as an alternative trade currency in the international markets (Tilly 1992). However, throughout the entire observation period, on average, 80% per cent of the credit lines provided by the bank were denoted in the sterling pound (Fig. 1); hence, the bank’s focus was the LMM. The *Brasilianische* accessed European money markets via three main channels: (i) the bank held current accounts at its mother institutions that provided unlimited access to capital denoted in German Marks, (ii) the *Disconto-Gesellschaft*’s London Office (which opened in 1901), and (iii) the bank’s London correspondents and agents gave access to the LMM (Supervisory Report Brasilianische, March 1888, Diouritch 1909) (Table 4).

**Table 4:** Correspondents and agents of the *Brasilianische* in London *Source* The Banking Almanac and Directory and The Brazilian Review

Bank	Years active*
Disconto-Gesellschaft	1901–1913
International of London Limited	1897–1904
N. M. Rothchild & Son	1898–1913
Union of London & Smith’s Bank Limited	1898–1913
William Brandt’s Sons & Co	1898–1913
Manchester and Liverpool District Banking Company Limited	1898–1913

We report 1897 because is the first year for which information is reported in the Banking Almanac. But even before 1897 the bank had correspondents in London

The world’s financial centre at that time was the City of London. In 1912, the *Brasilianische* Bank reported: ‘London is not only the largest, but commonly also the cheapest discount market in the world. […] Even today, one has to admit—it would be disingenuous not to—the first thing you need to start an overseas banking business is (…) a drawing address in London’^9^.

The sterling bill of exchange, a financial instrument issued worldwide, was the main trade tool on the LMM (Accominotti et al. 2021). These became a liquid, secure short-term borrowing option, irrespective of their connection to actual transactions. The bill’s validity hinged on the acceptor and endorser(s)’ signatures, offering returns based on the market discount rate influenced by market conditions and the Bank of England’s policy. The Bank’s pledge to unrestrictedly convert sterling notes to gold underpinned the sterling bill’s safety, solidifying its central role in the LMM^10^.

**Fig. 2:**
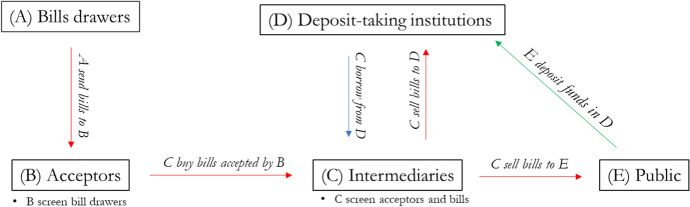
Functional roles in the London money market

Figure 2 provides a schematic representation of the functional roles of the actors in the LMM. Bills were drawn on London and sent there for acceptance. The role of acceptors was to screen the drawers of bills, i.e. to collect information on them and guarantee payment by accepting their bills. Once accepted, bills were bought by intermediaries, who had insider knowledge of the market and acted as screeners for acceptors and bills. These intermediaries usually did not hold the bills until maturity but endorsed them and re-sold them to deposit-taking institutions and the public. Deposit-taking institutions employed the funds collected among the public to make advances and buy bills from intermediaries. As a norm, they never rediscounted their bills but held them until maturity. Furthermore, deposit-taking institutions played another critical role in the LMM besides investing in bills. In fact, they also lent to the intermediaries most of the working capital they needed. Intermediaries borrowed short-term funds at the floating rate. When deposit-taking institutions had plenty of funds to lend, the floating rate was low, and vice versa.

Bill brokers and discount houses were unique intermediaries in the LMM acceptance market. Instead of costly direct engagement with merchant and individual acceptors, joint-stock banks outsourced bill scrutiny to these intermediaries (Clare 1896). They evaluated the bill’s price based on the borrower’s solvency and liquidity risk, understanding that market conditions affecting acceptor and drawer could change the price without impacting solvency. Bill brokers and discounting house agents would visit the offices of banks interested in buying bills (usually London joint-stock banks) and the offices of banks interested in selling bills (usually foreign and colonial banks) multiple times a day. This way, they obtained fresh, first-hand information that would otherwise be costly for joint-stock banks.

The market discount rate determined the price at which bills were bought and sold by bill brokers. The volume of bills the bill brokers transacted was a multiple of their capital, and they operated at leverage by borrowing short-term.^11^ While discount houses also collected deposits from the public, bill brokers relied exclusively on borrowing at the floating market rate from London banks and Anglo-foreign and foreign banks. As fragile as this system might seem, it proved remarkably stable. As King 1972, p.183) observed:

‘Then, as now, the call loan system rested upon the fundamental assumption that if one London banker were calling in loans, another banker would shortly receive a roughly equivalent amount, which he would seek to re-lend. In normal times, that was, and is, a warrantable assumption, and is the basic principle of deposit banking, as well as of bill dealing’.

In case of need, they could also sell their paper at a discount at the Bank of England—provided it was not a ‘Foreign Domicile’ or ‘Foreign Agency’ bill^12^. The floating rate hence determined how much leverage bill brokers and discount houses could take, and thus how large the turnaround of bills would be. When the discount rate was relatively high compared to the floating rate, bill brokers had all the incentives to move large amounts of bills. Furthermore, if market conditions were particularly favourable, they could even hold some bills until maturity. De facto*,* they would do maturity transformation by borrowing at the floating rate, and lending at the market discount rate. Therefore, when the spread between the discount and the floating rate was large it was easier for international banks to place their bills on the LMM. Another even more straightforward mechanism through which the spread could affect the demand for foreign bills was taking the perspective of the deposit-taking institutions. If the floating rate was low, it meant that these banks had an abundance of funds to employ. If the discount rate was high, it implied that investing in bills was more remunerative. Therefore, a large spread indicates that discounting bills was particularly profitable, relative to the liquidity of deposit-taking institutions.

**Fig. 3:**
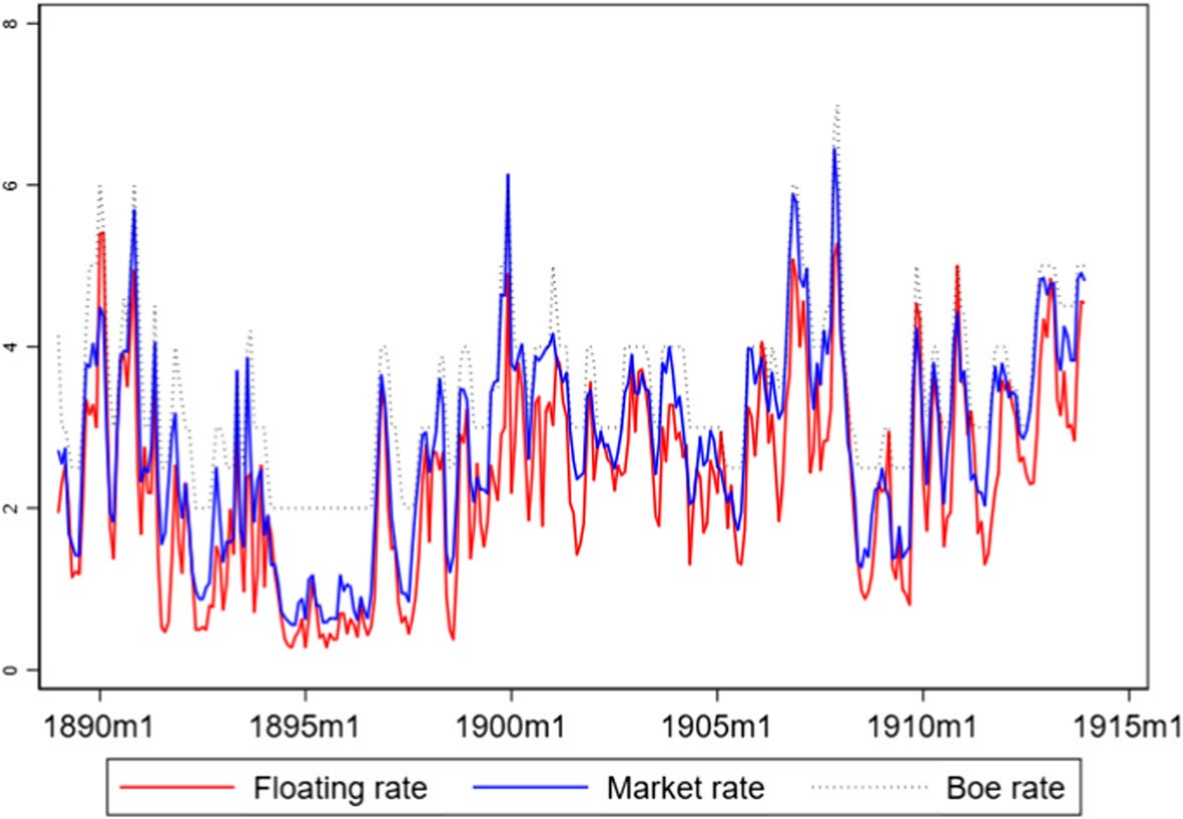
Floating, market, and BoE rates (1889–1913)

**Fig. 4:**
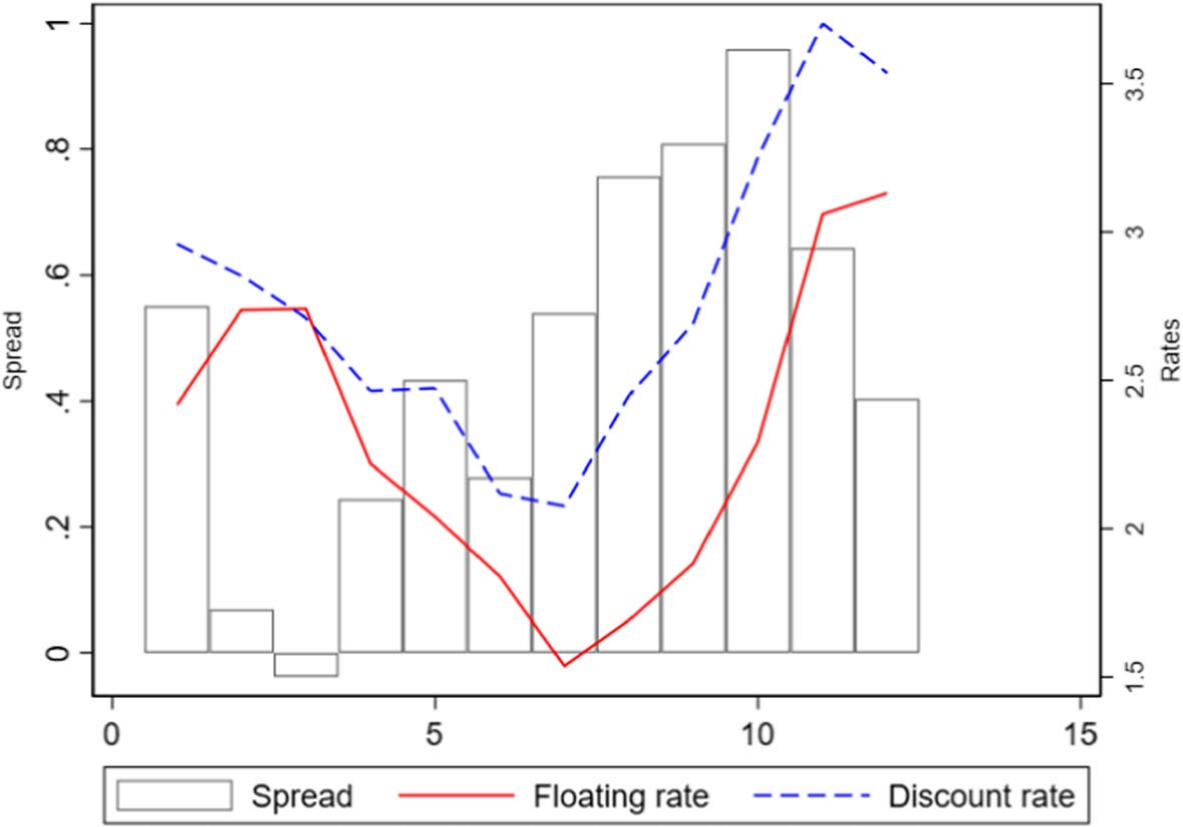
Market rate, floating rate and spread in London (monthly averages)

Figure 3 illustrates the fluctuations between the floating rate and the market rate against the backdrop of the Bank of England’s rate from 1890 to 1913. These fluctuations reflect the liquidity conditions in the LMM. A higher floating rate compared to the market rate suggests periods of liquidity shortage, prompting lenders to charge more for short-term funds. Conversely, a lower floating rate indicates liquidity surpluses, making it cheaper to borrow. The Bank of England’s rate acts as a benchmark, influencing these rates and signalling monetary policy changes. Contemporary commentaries acknowledged that the difference between the two rates reflected general liquidity conditions in the market. On Friday 19th October, floating rate was 1.5%, the market rate 3.75, and the BoE rate 4%. The ‘Discount and Loan Market’ section of the Economist commented: ‘money has remained plentiful’^13^. By contrast, on Friday 9th March, day-to-day loans were 3.5%, market rate 4%, and BoE rate 4%—the Economist noted that ‘Money has grown scarcer’^14^. Similarly, in October 1903, the Monetary Review of the Bankers’ Insurance Managers’ and Agents’ Magazine, described the September situation with the floating rate at 3.5 and the market rate at 4% as stringent. By contrast, the following month, when the floating rate was 1.75 and the market rate 3.65, the subtitle of the Monetary Review section was ‘easier money’^15^. As shown in Fig. 4 (Sect. 4), the spread between the two rates is usually higher in autumn and lower in March. Peake (1926) observed these trends and explained that they were due to a greater supply of bills in autumn, necessitated by the financing of crop shipments after summer harvests. This situation affected the market rate more significantly than the floating rate. Conversely, February and March are quieter months for trade. Therefore, the stringent effect of the floating rate is not transferred to the market rate, as the supply of bills is low during this period.

## Empirical Strategy: Credit Provision and the London Money Market

In this section, we empirically investigate the relationship between the London spread of market and floating rate (London spread) and the monthly amount of credit authorised by the *Brasilianische* Bank between 1889 and 1913. Our main hypothesis is that the London spread positively affects the bank’s monthly credit lines. Expressed in the form of equation, the model is as follows:

1 $$X_{{\text{m}}} = \alpha_{0} + \beta {\text{LonSpread}}_{{\text{m}}} + \Upsilon_{{\text{m}}} + \lambda_{{\text{y}}} + \lambda_{{\text{m}}} + \varepsilon_{{\text{t}}}$$

where *X*_m_ is the total value in sterling pounds of credit lines authorised by the *Brasilianische* in month *m*, *α*_0_ is a constant. For this study, the key coefficient of interest is *β*, which shows the impact of the London spread on the credit lines of the *Brasilianische*. **ϒ**_m_ is a set of control variables accounting for the different influences on the credit provision of the *Brasilianische* and/or the LMM conditions. The estimated model also includes year (*λ*_y_) and month (*λ*_m_) fixed effects. We use ordinary least squares (OLS) to estimate Eq. 1, and we employ Newey–West standard errors to account for potential autocorrelation and heteroskedasticity.

We take the data on monthly credit provided by *Brasilianische* Bank from 1889 until 1913 from Kisling (2020). The data come from the official reports of *Brasilianische* Bank’s supervisory board (Aufsichtsratberichte)^16^. The supervisory board of *Brasilianische Bank* held its meetings three or four times annually at the bank’s headquarters in Hamburg, Germany. In these gatherings, the supervisory board determined the monthly credit lines for all branches of the *Brasilianische Bank*^17^, valid until the subsequent meeting. The duration of each credit line was at least the time between two board meetings, which, although variable yearly, was never less than one month. In the reports, financing was differentiated into: (i) lines of direct credit and (ii) the maximum value for the discount of bills of exchange. For this study, we are interested exclusively in the credit lines and bills of exchange denoted in sterling pounds. The monthly floating rate in London was taken from Nishimura (1971), who retrieves the data from the Economist. London’s Monthly Market Discount Rate is taken from the NBER Macrohistory Database. For our econometric analysis, we test for the possible influence of a series of additional variables on the monthly credit authorised by the *Brasilianische.* Unless otherwise stated, we retrieved the information for these independent variables from the NBER Macrohistory Database^18^. For reasons of clarity, we maintained the original names of the variables as indicated in the source, which we report in *italics.* Table 12 in the Appendix shows the descriptive statistics of all variables employed. We test for the stationarity of the dependent and independent variables using the augmented Dickey-Fuller test. We use 12 lags to account for monthly data. Table 13 in the Appendix presents the results. At the 10% level, we can always reject the hypothesis that the dependent variable, our variable of interest, and our main control variables are non-stationary. They are thus considered I(0).

We include four main controls in our baseline regression. The variable ‘spread between discount and call money rates in New York’ quantifies the difference between the *Commercial Paper Rate for New York* and the *Call Money Rate for USA*. Its relevance stems from the US’s major trade partnership with Brazil and New York’s position as a significant financial centre, potentially influencing the dynamics of the LMM. The variable ‘Mark/$ exchange rate’ represents the *Average Monthly Berlin Rate of Exchange on New York for Germany*. It controls for fluctuations in the foreign exchange rate that might affect the investment decisions of the *Brasilianische* Bank. Furthermore, given the substantial involvement of German banks in the LMM, fluctuations in the value of the Mark can exert a notable influence on the dynamics of the LMM. The ‘spread between Reichsbank and Bank of England (BoE) official rates’ is the difference between the *Official Bank Discount Rate for Germany* and the *Bank of England Policy Rate in the United Kingdom*. Interest rate differentials between Britain and Germany could drive gold movements closely linked to LMM fluctuations. Additionally, this spread might influence credit limits in £, with the bank favouring credit lines in Marks if advantageous^19^. Finally, we consider the variable ‘Trade Union Members Unemployed in UK, % (log)’, which is the *Trade Union Members Unemployed, Total for United Kingdom*. It serves as an indicator of the state of the British economy, which at 8.22% of the global GDP in 1913 could have a significant impact on the LMM and the Brazilian economy^20^.

Table 5 presents the results of our baseline regression (1). They confirm our main hypothesis. The coefficient of the London spread is positive and significant, confirming a positive correlation between the difference between the market and floating rate in London and the monthly amount of credit authorised by the bank. The effect is sizeable. A one-unit increase in the spread is associated with an average rise in monthly-authorised credit by 64,273 pounds, which is ~ 5% and ~ 20% of the mean and the standard deviation of our dependent variable, respectively (column 4).

**Table 5:** Results regression estimations—baseline model—Eq. (1)

	(1) OLS	(2) OLS	(3) OLS	(4) OLS
Spread between discount and floating rates	75.767***	65.801***	71.054***	64.273***
	(23.170)	(24.535)	(18.782)	(20.897)
Spread between discount and call money rates, New York			19.959**	18.435*
			(9.722)	(10.194)
Mark/$ exchange rate			− 1,685.396***	− 1,468.660**
			(499.661)	(601.113)
Spread between Reichsbank and BoE official rates			− 26.927**	− 23.289*
			(13.396)	(12.642)
Trade Union Members Unemployed in UK, % (log)			− 93.210**	− 53.216
			(39.440)	(44.533)
Constant	290.345***	218.145***	7,404.490***	6,406.049**
	(12.471)	(27.574)	(2,090.060)	(2,539.114)
Observations	300	300	300	300
Month indicators	No	Yes	No	Yes
Year indicators	Yes	Yes	Yes	Yes
Controls	No	No	Yes	Yes
adjR2	0.825	0.827	0.830	0.829
*F* test model	873.2	1427	492.5	455.1
*P*-value of *F* model	0	0	0	0

The dependent variable is the total monthly credit authorised by the *Brasilianische* between 1889 and 1913 in thousand £. Standard errors are estimated with Newey–West and reported in parentheses

***p < 0.01, **p < 0.05, *p < 0.1

Column (1) shows the coefficient of London spread with only yearly indicators; Column (2) includes monthly and yearly indicators. Columns (3) and (4) add our main controls using only year and year and month indicators, respectively. Including monthly indicators ensures that seasonal patterns do not drive our results. The comparison of the results of our estimations with and without month indicators shows that seasonality is not likely to be the main driver of the correlation we find. In fact, the coefficient of the London spread is only marginally smaller when including monthly indicators. This is especially true in our models (columns 3 and 4), which include our main control variables.

To test the robustness of our findings, we run our estimations with two additional sets of control variables. Among these, only some variables are stationary at I(0), while others are at the I(1) level. In the latter case, we take the first differences (see Table 13 in the Appendix). If not otherwise stated, the information for these variables are taken NBER Macrohistory Database. The results are presented in Tables 6 and 7, respectively. They confirm our main hypothesis, with the size and significance of the coefficient of London spread being consistent.

**Table 6:** Results regression estimations—OLS—Eq. (1)—including set of additional control variables

	(I)	(II)	(III)	(IV)	(V)	(VI)
Spread between floating and discount rates	67.626***	62.749***	70.622***	63.972***	71.731***	64.524***
	(18.813)	(21.402)	(18.594)	(21.009)	(23.113)	(20.453)
Spread between Reichsbank and BoE official rates	− 26.893**	− 24.130*	− 25.207*	− 21.690*	− 26.591**	− 24.528*
	(13.416)	(12.845)	(13.001)	(12.696)	(13.184)	(12.748)
Trade Union Members Unemployed in UK, % (log)	− 86.797**	− 47.746	− 87.403**	− 49.015	− 89.067*	− 50.565
	(40.813)	(46.852)	(40.359)	(44.840)	(45.852)	(44.071)
Mark/$ exchange rate	− 1,826.000***	− 1,548.534**	− 1,805.641***	− 1,570.491**	− 1,829.448***	− 1,570.000**
	(504.998)	(614.893)	(510.502)	(612.729)	(491.270)	(620.751)
Spread between discount and call money rates, New York	19.570**	19.523*	21.479**	20.593*	20.936**	18.941*
	(9.279)	(10.688)	(10.190)	(11.141)	(10.446)	(10.566)
Total Imports for Great Britain, mln £ = *D*,	2.546	3.790				
	(2.206)	(3.523)				
Total Exports of Produce and Manufactures for Great Britain, mln £ = *D*,			2.967	6.687*		
			(2.313)	(3.413)		
Total exported bags of coffee from Rio de Janeiro (log)					− 9.303	0.790
					(27.339)	(40.268)
Price of coffee (log) £ = *D*,					260.514	310.023
					(237.702)	(237.309)
Constant	8,000.697***	6,749.458***	7,913.535***	6,832.133***	8,128.750***	6,822.006**
	(2,107.315)	(2,595.265)	(2,130.793)	(2,587.250)	(2,066.123)	(2,697.258)
Observations	299	299	299	299	299	299
Month indicators	No	Yes	No	Yes	No	Yes
Year indicators	Yes	Yes	Yes	Yes	Yes	Yes
Controls	Yes	Yes	Yes	Yes		
adjR2	0.825	0.824	0.825	0.824	0.825	0.824
*F* test model	639.7	421.4	606.4	454.3	470.5	333
*P*-value of *F* model	0	0	0	0	0	0

	(VII)	(VIII)	(IX)	(X)	(XI)	(XII)
Spread between floating and discount rates	68.890***	61.136**	58.242***	55.844*	58.717***	54.198**
	(18.671)	(24.637)	(17.318)	(28.671)	(16.768)	(25.103)
Spread between Reichsbank and BoE official rates	− 25.532	− 22.832	− 23.238	− 22.877	− 23.685	− 23.369
	(21.841)	(20.548)	(22.396)	(21.477)	(16.357)	(15.049)
Trade Union Members Unemployed in UK, % (log)	− 87.193*	− 50.303	− 109.562**	− 69.587	− 107.193***	− 68.374
	(47.865)	(47.143)	(45.902)	(46.157)	(39.483)	(48.532)
Mark/$ exchange rate	− 1794.891**	− 1500.934*	− 1719.048**	− 1544.421*	− 1622.508***	− 1507.759**
	(706.150)	(853.931)	(733.403)	(878.192)	(563.611)	(701.864)
Spread between discount and call money rates, New York	21.080**	18.859	19.667*	17.635	19.517*	17.798
	(10.385)	(11.722)	(10.556)	(12.331)	(10.789)	(11.798)
Pig Iron Output for Germany, ‘000 metric tons = *D*,	− 0.137	− 0.222				
	(0.143)	(0.233)				
Clearings of Reichsbank for Germany, bln Marks = *D*,	10.654	21.507				
	(19.340)	(29.271)				
Earnings of Prussian − Hessian Railways from Freight for Germany, mln Marks = *D*,	0.801	1.458				
	(0.968)	(1.221)				
Weights of Exports for Germany, ‘000 metric tons = *D*,			0.006	− 0.014		
			(0.016)	(0.018)		
Weights of Imports for Germany, '000 metric tons = *D*,					0.012	0.005
					(0.008)	(0.005)
Constant	7869.410***	6546.134*	7984.943***	7125.639*	7576.415***	6982.474**
	(2950.835)	(3597.390)	(3054.401)	(3684.378)	(2349.754)	(2959.489)
Observations	299	299	273	273	273	273
Month indicators	No	Yes	No	Yes	No	Yes
Year indicators	Yes	Yes	Yes	Yes	Yes	Yes
Controls	Yes	Yes	Yes	Yes	Yes	Yes
adjR2	0.824	0.822	0.664	0.661	0.665	0.660
*F* test model	158.4	146.7	111.1	77.48	315	221.4
*P*-value of *F* model	0	0	0	0	0	0

***p < 0.01, **p < 0.05, *p < 0.1

The dependent variable is the total monthly credit authorised by the *Brasilianische* between 1889 and 1913 in thousand £. Standard errors are estimated with Newey–West and reported in parentheses. The regressions are estimated with ordinary least squares (OLS)

**Table 7:** Results regression estimations—OLS—Eq. (1)—including set of additional control variables

	(I)	(II)	(III)	(IV)	(V)	(VI)	
Spread between floating and discount rates	71.091***	64.401***	71.294***	63.866***	71.648***	64.503***	
	(19.263)	(20.525)	(18.975)	(20.943)	(19.307)	(21.488)	
Spread between Reichsbank and BoE official rates	− 26.217**	− 22.789*	− 25.568*	− 22.678*	− 26.248**	− 23.151*	
	(13.084)	(12.645)	(13.499)	(13.102)	(13.022)	(12.741)	
Trade Union Members Unemployed in UK, % (log)	− 92.623**	− 54.150	− 86.935**	− 50.092	− 89.984**	− 52.845	
	(40.588)	(44.623)	(40.853)	(45.078)	(41.171)	(46.005)	
Mark/$ exchange rate	− 1,819.396***	− 1,468.153**	− 1,734.161***	− 1,466.511**	− 1,806.717***	− 1,520.757**	
	(522.510)	(634.905)	(501.548)	(597.937)	(518.249)	(633.025)	
Spread between discount and call money rates, New York	22.035**	20.609*	21.007**	18.877*	21.031**	18.991*	
	(9.784)	(10.889)	(10.049)	(10.583)	(9.945)	(10.594)	
Security Price Index for London = *D*,	7.362	14.249*					
	(7.328)	(8.231)					
Milreis/$ exchange rate = *D*,			− 19.725	− 18.668			
			(33.313)	(33.352)			
Rubber prices = *D*,					35.814	35.649	
					(90.801)	(79.654)	
Constant	7972.163***	6397.796**	7613.656***	6401.016**	7918.987***	6629.293**	
	(2181.380)	(2679.860)	(2094.727)	(2525.612)	(2163.191)	(2671.501)	
Observations	299	299	299	299	299	299	
Month indicators	No	Yes	no	Yes	No	Yes	
Year indicators	Yes	Yes	Yes	Yes	Yes	Yes	
Controls	Yes	Yes	Yes	Yes	Yes	Yes	
adjR2	0.825	0.824	0.825	0.823	0.824	0.823	
*F* test model	585.4	465.1	620.8	503.7	581.9	465.9	
*P*-value of *F* model	0	0	0	0	0	0	

	(VII)	(VIII)	(IX)	(X)	(XI)	(XII)	(XIII)	(XIV)
Spread between floating and discount rates	70.967***	69.802***	78.049***	68.988***	72.774***	57.479***	72.372***	60.604***
	(19.397)	(21.583)	(19.641)	(21.532)	(21.554)	(22.115)	(21.980)	(22.965)
Spread between Reichsbank and BoE official rates	− 26.995**	− 15.333						
	(13.346)	(11.800)						
Trade Union Members Unemployed in UK, % (log)	− 92.775**	− 79.215*	− 81.783**	− 46.145	− 77.211*	− 9.214	− 73.548*	− 16.186
	(42.161)	(46.358)	(40.803)	(45.143)	(44.685)	(40.581)	(42.631)	(40.262)
Mark/$ exchange rate	− 1679.522***	− 1746.033***	− 2075.143***	− 1731.185***				
	(540.846)	(665.038)	(536.498)	(583.098)				
Spread between discount and call money rates, New York	19.831*	22.300*	20.633**	19.147*	8.629	6.423	7.901	8.250
	(11.912)	(11.573)	(9.839)	(10.617)	(10.485)	(11.462)	(11.938)	(12.710)
Spread between German official and market discount rate	− 1.034	81.052						
	(28.949)	(57.326)						
Spread between market discount rate in London and Berlin = *D*,			− 3.342	8.632				
			(9.231)	(11.163)				
Official discount rate Reichsbank					9.628	40.552**		
					(17.792)	(16.984)		
Market discount rate in Berlin							8.755	16.920
							(17.190)	(19.075)
Constant	7380.768***	7450.166***	9040.184***	7502.832***	305.365***	58.716	315.820***	187.751***
	(2252.000)	(2788.637)	(2238.832)	(2461.935)	(54.506)	(69.514)	(44.144)	(46.357)
Observations	300	300	299	299	300	300	300	300
Month indicators	No	Yes	No	Yes	No	Yes	No	Yes
Year indicators	Yes	Yes	Yes	Yes	Yes	Yes	Yes	Yes
Controls	Yes	Yes	Yes	Yes	Yes	Yes	Yes	Yes
adjR2	0.830	0.832	0.824	0.822	0.826	0.830	0.826	0.827
*F* test model	486.8	268.8	750.5	537.3	1321	2021	1372	1968
*P*-value of *F* model	0	0	0	0	0	0	0	0

***p < 0.01, **p < 0.05, *p < 0.1

The dependent variable is the total monthly credit authorised by the *Brasilianische* between 1889 and 1913 in thousand £. Standard errors are estimated with Newey–West and reported in parentheses. The regressions are estimated with ordinary least squares (OLS)

Since the primary function of the LMM was to finance international trade and the *Brasilianische* Bank did not only finance German trade, but also British firms, controlling for British imports and exports is crucial. We hence include in our first control set: ‘Total Imports for Great Britain, mln £ (difference)’, which is the first difference of *Total Imports, Value for Great Britain*; ‘Total Exports of Produce and Manufactures for Great Britain, mln £ (difference)’, which is the first difference of *Total Exports of Produce and Manufactures for Great Britain*. To control for variations in Brazilian coffee exports, we include the ‘Total exported bags of coffee from Rio de Janeiro (log) (difference)’, the log value of the number of bags of 60 kg exported every month from Rio de Janeiro^21^. As the principal export commodity of Brazil, coffee had the potential to influence the lending of the *Brasilianische*. We furthermore account for possible demand shocks to the Brazilian economy caused by changes in the price of coffee with the variable ‘Price of coffee (log) £’, that is, *Brazil Santos Arabicas Spot Price (Cents/Pound) (with GFD Extension)*^22^.

These controls are particularly important because we cannot include monthly indicators in our IV specification (see next section). Thus, including these variables should guarantee that we at least control for the seasonal fluctuations that should worry us the most—those in the coffee market. Finally, we test for the possible influence of the German business cycle. The *Brasilianische* funded trade between Germany and Brazil, and its lending likely depended on the macroeconomic conditions at home. At the same time, Germany was the second largest European economy^23^, and therefore its macroeconomic fluctuations inevitably could impact the LMM. We account for these influences with the following variables: ‘Pig Iron Output for Germany, ‘000 metric tons (difference)’ is *Pig Iron Output for Germany*; ‘Clearings of Reichsbank for Germany, bln Marks (difference)’ is *Clearings of Reichsbank for Germany*; ‘Earnings of Prussian-Hessian Railways from Freight for Germany, mln Marks (difference)’ is *Earnings of Prussian-Hessian Railways from Freight for Germany*; ‘Weights of Imports for Germany, ‘000 metric tons (difference)’ is *Total Imports—Weight for Germany*; ‘Weights of Exports for Germany, ‘000 metric tons (difference)’ is *Exports, Total, Weight for Germany*.^24^ Furthermore, in Table 13, we also control for imports and exports of the USA and France two other important trade partner of Brazil^25^.

In our second set of control variables, Table 7, we test for the possible influence of market conditions in Germany, dynamics in the British capital market, and fluctuations in Brazilian exchange rates. The ‘Spread between German official and market discount rate’ is the difference between the ‘Market discount rate in Berlin’, which is the *Private Discount Rate, Prime Banker’s Acceptance, Open Market for Berlin, Germany,* and the ‘Official discount rate Reichsbank’, which is the *Official Bank Discount Rate for Germany.* This spread serves as a crucial indicator of the tightness of credit conditions in Germany. As highlighted by Bignon et al. (2012), a negative spread can signal that a Central Bank is rationing credit. Conversely, when the market rate significantly dips below the official rate, it implies an abundance of liquidity in the market, suggesting that banks do not rely heavily on Central Bank financing. Given that the *Brasilianische Bank*, headquartered in Hamburg, operates as a German bank, variations in the German rates and liquidity conditions can significantly influence its lending strategies. Moreover, considering Germany’s status as the second largest European economy after Britain, it is reasonable to presume that exogenous variations in German rates and market conditions could likewise impact the LMM. The variable ‘Spread between market discount rate in London and Berlin’ is the difference between *Open Market Rates of Discount for London, Great Britain* and *Private Discount Rate, Prime Banker’s Acceptance, Open Market for Berlin, Germany*. The rationale for their inclusion in the model is the same as for the ‘Spread between Reichsbank and BoE official rates’. ‘Security Price Index for London (difference)’ is *Security Price Index for London, Great Britain*. We control this variable to account for dynamics in the British capital market. While the effect on the LMM is straightforward, since many stock brokers borrowed short-term on the Money Market, we are concerned that a surging capital market could provide alternative investment opportunities for the *Brasilianische* Bank. We include ‘Milreis/$ exchange rate (difference)’ which is *Brazil Real per US Dollar (with GFD Extension)* to account for exchange rate fluctuations.^26^ Lastly, it is essential to consider the heavy dependence of the Brazilian economy on rubber exports. A sudden shift in rubber prices could induce a demand shock within the Brazilian economy, potentially leading to repercussions in the LMM. To account for this, we include the variable ‘Price of rubber (log) $’ is Rubber Spot Price (USD/Kilogram) (with GFD Extension).^27^

Our robustness checks reveal that none of these variables significantly alters the size and significance of the London spread coefficient. Demand shocks to the Brazilian economy, reflected through variables such as the total exported bags of coffee from Rio de Janeiro and the price of coffee, do not seem to exert substantial influence on the key relationship under study. This implies that factors intrinsic to the Brazilian economy, such as fluctuations in the coffee trade or the price of rubber, do not significantly interfere with the primary dynamics we are studying.

Likewise, variables capturing the British trade, German business cycle, and German trade do not affect our key findings. Although these controls are integral to understanding the lending activities of the *Brasilianische Bank* and the macroeconomic conditions of these economies, they do not seem to change the central relationship being investigated. Similarly, variables related to the German discount rates, the spread between German official and market discount rate, the spread between the market discount rate in London and Berlin, the British capital market and the Brazilian exchange rate also fail to substantiate a significant shift in the size and significance of the London spread coefficient. This demonstrates that although these factors are vital to their respective economies, their influence does not materially alter our core findings. In sum, these robustness checks confirm the stability of our results.

Finally, we conducted an additional set of robustness checks on our main explanatory variable, the London spread. The board meetings did not occur every month and were obviously not random. It is likely that the values of the spread between meetings influenced the decisions taken at these meetings. We introduced three alternative specifications of our London spread. The first specification takes the average of the spread between two meetings. The second specification carries forward the value of the spread of the last meeting until the following one. The third specification takes the average of the spread between *t*, *t*-1, and *t*-2. Our results remain robust, with our coefficient continuing to be large and significant. These results are displayed in Table 14.

## IV approach: credit provision and tax revenues

Our OLS regression analysis should not suffer from reverse causality between our dependent and independent variables. It seems quite implausible that a German foreign bank in Brazil had the capacity and market power to influence the European money market. However, despite the comprehensive robustness checks affirming our results, the possibility of omitted variable bias cannot be entirely disregarded. To mitigate potential endogeneity concerns, we are employing an instrumental variables (IV) approach, informed by our historical understanding of the functioning of the LMM.

Our IV is based on the historical effect of British tax revenues on the LMM.^28^ The tax revenue collection in Great Britain at that time took place between February and April. During this period, British firms and individuals had to withdraw their money from London joint-stock banks to pay income and other taxes. The decrease in bank deposits resulted in the reduction of floating money, causing a contraction in short-term liquidity due to banks having fewer funds to extend as loans. This led to an increase in the floating interest rate from the end of February until the beginning of April, and consequently a decrease in the spread. We do not capture monthly tax payments. However, the tax payments were made directly to the Bank of England, leading to an increase in their public deposits. We use the fluctuations of these public accounts as IV^29^. Figure 8 in the Appendix reflects this relationship, illustrating the inverse correlation between the spread and the proportion of public deposits at the Bank of England (BoE).

The exogeneity of our IV derives from several factors. Firstly, it is based on the premise that the impact of British tax collection is confined to the banking sector within Great Britain itself^30^. The resultant fund transfers to the BoE’s Government account could only indirectly affect the Brasilianische’s credit lines through the LMM spread. Even the opening of a London branch by one of the mother institutions of the Brasilianische after 1901 (Disconto-Gesellschaft) would likely have had a limited impact. Typically, London branches of foreign banks didn’t collect a significant portion of domestic deposits, but rather focused on acceptances. It is hence highly unlikely that they had a significant amount of British customers that were subject to paying taxes and thus would withdraw their deposits. Secondly, there was the potential for unpredictable variations in the timing of tax payments. The income tax, being the primary tax collected, could be paid in two instalments—in March and July. While the bulk of it was typically paid in March, uncertainty remained for the bank regarding whether the entire amount would be paid in March or partly in July. Thirdly, the total income tax collected was not necessarily linked to the performance of the British economy. As depicted in Fig. 9 Appendix, the total income tax showed little correlation with GDP growth during the period under review. In fact, income tax rates experienced several changes during this time (Seligman, 1911). This potential unpredictability further strengthens our argument for the exogeneity of the instrumental variable (IV).

Figure 4 displays the monthly average for the floating rate, the London market discount rate, and the spread between the two. It showed an evident contraction of the spread around March when the floating rate increased, and the market rate continued its trend. Contemporaries widely acknowledged this phenomenon.^31^

In his *Academic study of the London money market,* Peake (1926, p.9) displayed precisely the graph we plotted in Fig. 4 and commented ‘the rise in the floating rate to March is due, of course, to the collection of the taxes at the end of the financial year’. In his seminar study, *The London Money Market,* Spalding (1922, p.77) identified four periods of fluctuations in the LMM:^32^

‘This brings us to the second period, one in which the market is largely under the shadow of the tax-gatherers’ demand. Owing to the ingathering of revenue, stringent conditions are usually expected and experienced towards the end of the Government’s financial year in March. In fact, it is in March that the balances of the Chancellor of the Exchequer with the Bank of England reach their high-water mark and, as the money is kept off the market for a time, those who require accommodation have to pay high rates for it’.

**Fig. 5:**
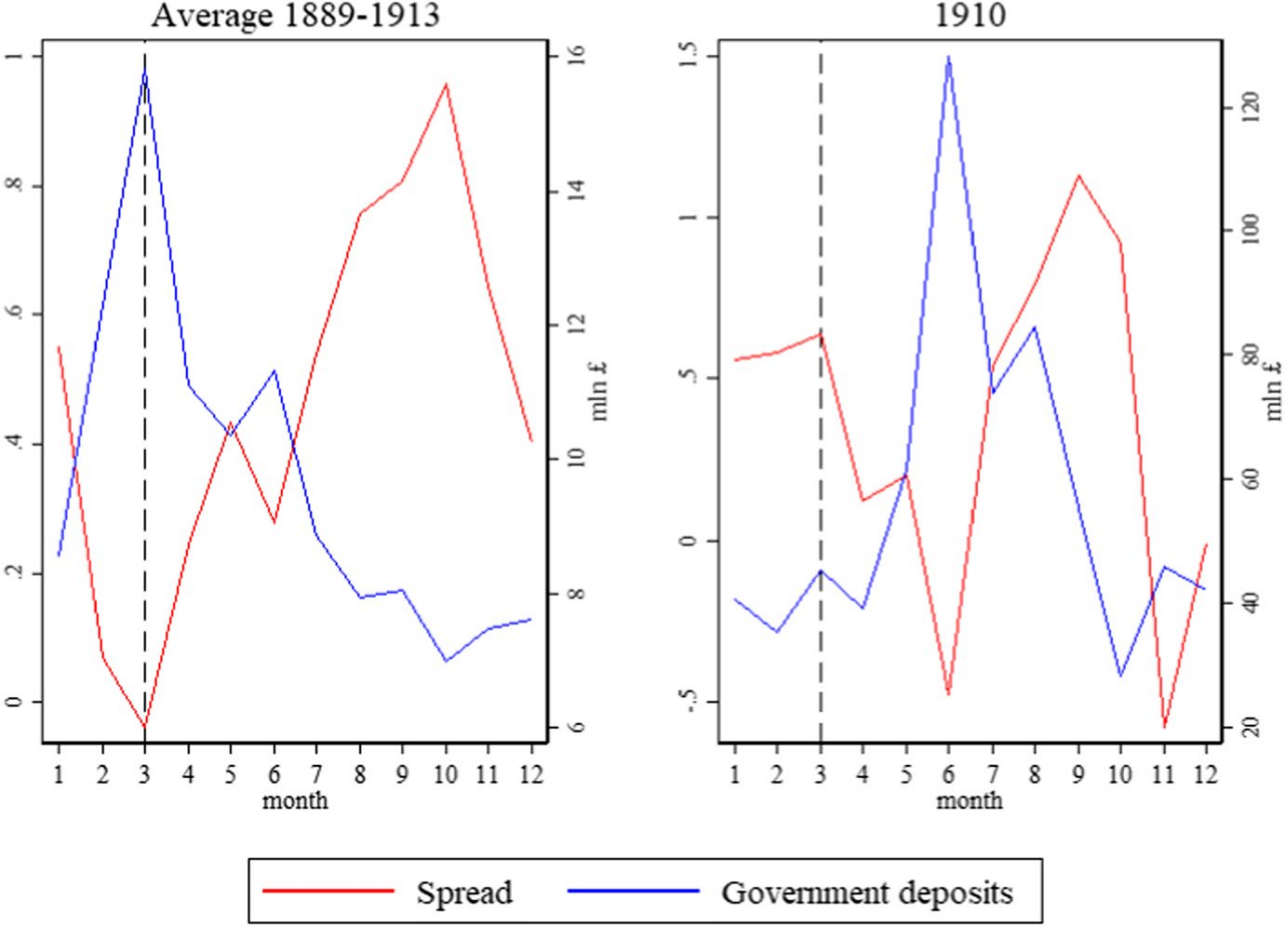
London spread and Government deposits at BoE—Average 1889–1913 and 1910

**Fig. 8:**
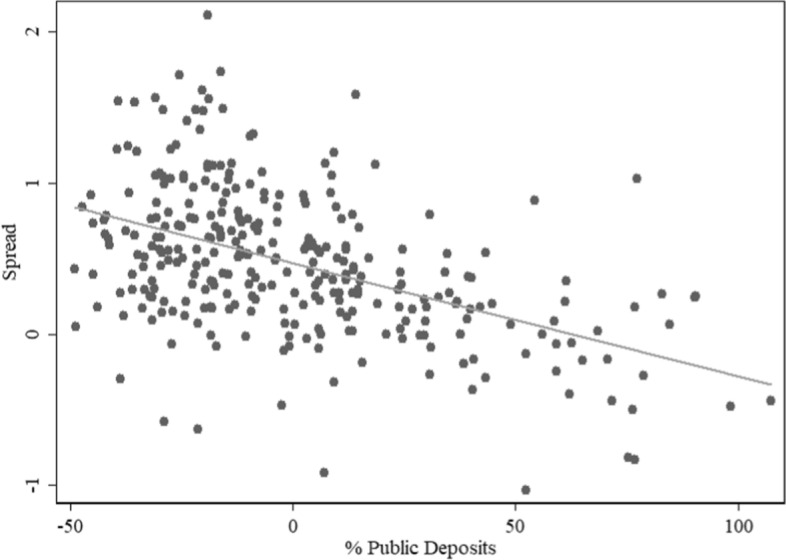
Correlation between spread of the London market and floating rate and the percentage change in public deposits at the BoE, 1889–1913. *Source* The spread represents the difference between the monthly floating rate and the monthly market discount rate in London. The monthly floating rate was obtained from Nishimura (1971), who sourced the data from the economist. London’s monthly market discount rate was obtained from the NBER Macrohistory Database. Data on BoE public deposits are taken from Huang and Thomas (2016)

**Fig. 9:**
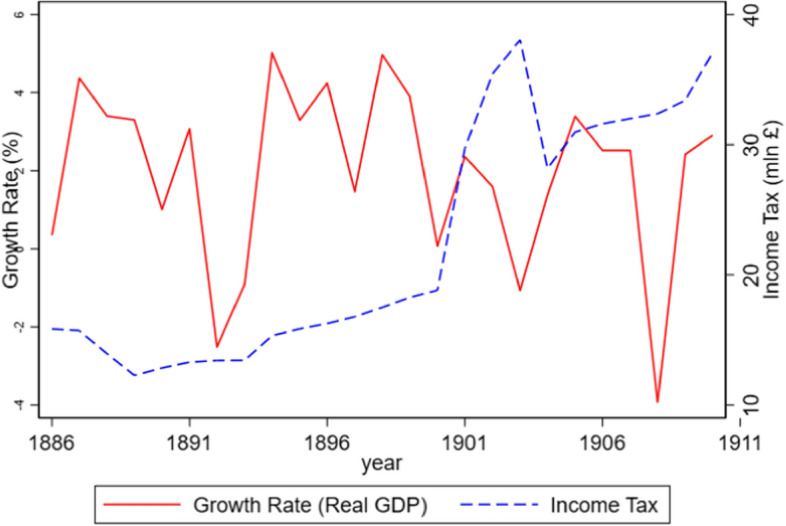
Annual British growth rate (GDP) and income tax (1886–1910)

**Fig. 10:**
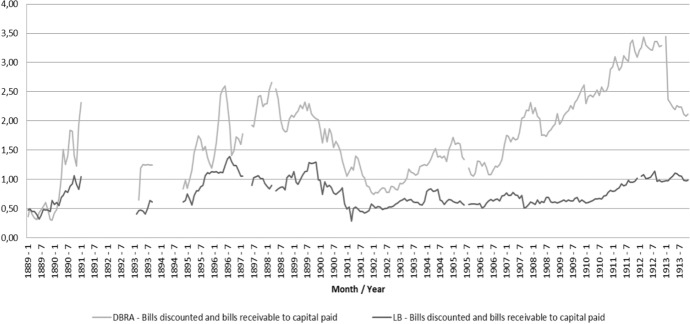
Ratio of bills discounted and bills receivable to capital paid in—Brasilianische Bank für Deutschland and London & Brazilian Bank de Rio de Janeiro—monthly relation, 1893–1913. *Source* Kisling (2017), page 198

**Fig. 11:**
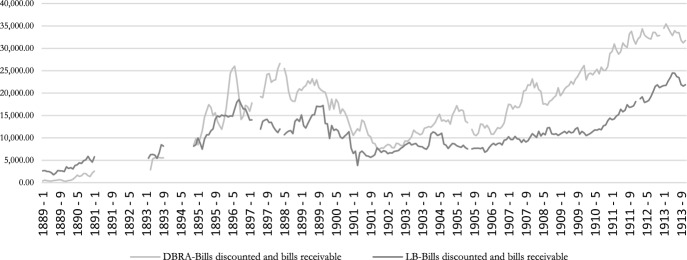
Bills discounted and bills receivable—Brasilianische Bank für Deutschland and London & Brazilian Bank de Rio de Janeiro—1893–1913 (Figures in Milreis). *Source* own elaboration based on: The Brazilian Review, O Commercio de Sao Paolo, Jornal do Comercio Retrospecto, The Rio News, various years

One challenge our IV faces is the seasonality of the tax collection. It always happens during the same months of the year. However, the inclusion of monthly indicators in our estimation would absorb much of the correlation between our spread variable and our instrument. At the same time, the results of our OLS estimation show (Table 1) that the correlation between our dependent variable and the spread holds well with and without including monthly indicators, suggesting that seasonality should be a minor concern. However, a priori, we cannot rule out that the squeeze in the spread and the collection of taxes are a spurious correlation that depends on unobserved seasonal factors. We could not find any trace of such unobservable in the coeval press, but, fortunately, the constitutional crisis that followed the People Budget of 1909 allows us to dispel any doubt. In 1909, Lloyd George proposed a fiscal Budget with substantial progressive measures, including a land tax and a ‘super tax’ (or surtax) to be levied on incomes over £5000. The House of Lords vetoed the proposal, and new general elections were called. The Finance Bill was finally approved only in April 1910, and income tax collection for that year took place in May and June rather than in March. Figure 5 shows that the London spread and Government deposits typically show a robust opposite dynamic in February–March. In 1910, when tax collection took place in May–June, the same pattern emerged but shifted by three months. The absence of any pattern in February–March 1910 confirms that the strong divergence we observe in other years can be safely attributed to tax collection and not to unobserved seasonal patterns. Therefore, estimating our IV without month indicators should not be a problem.

We use a 2SLS approach for our IV estimation. In the form of an equation, our model is expressed as follows:

2 $${\text{LonSpread}}_{{\text{m}}} = \alpha_{0} + \zeta TR_{{\text{m}}} + \Upsilon_{{\text{m}}} + \lambda_{{\text{y}}} + \varepsilon_{{\text{m}}}$$

3 $$X_{{\text{m}}} = \alpha_{0} + \beta \widehat{{{\text{LonSpread}}_{{\text{m}}} + }}\Upsilon_{{\text{m}}} + \lambda_{{\text{y}}} + \varepsilon_{{\text{m}}}$$

with (Corr (TR_m_ *ɛ*_m_) = 0).

Here, LonSpread is the spread between the London market and floating rate in month *m*. $\widehat{{\text{LonSpread}}}$ is the predicted value of LonSpread. TR_m_ serves as our instrumental variable (IV), representing the percentage difference between the mean monthly public deposits at the Bank of England (BoE) and the mean annual public deposits at the BoE, measured relative to the mean annual public deposits at the BoE. *λ*_y_ are year indicators. The rest of the specifications of Eq. (3) are identical to regression (1). Table 8 compares the results of our OLS baseline model with the results of our IV estimation. They confirm our previous findings. The coefficient of our main variable of interest, the London spread, is positive and significant. Our IV coefficients are larger than our OLS coefficients, but this should not be due to a weak instrument problem. The first stage IV *F* statistics (reported in Table 8) confirms that our instrument is very strongly correlated with our dependent variable, and thus we can rule out the issue of a weak instrument.

**Table 8:** Results regression estimations—IV model—Eqs. (1) and (3)

	(1) OLS	(2) IV	(3) OLS	(4) IV
Spread between floating and discount rates	75.767***	95.472**	71.054***	97.032**
	(23.170)	(43.448)	(18.782)	(46.916)
Spread between discount and call money rates, New York			19.959**	16.657*
			(9.722)	(8.691)
Mark/$ exchange rate			− 1685.396***	− 1799.487***
			(499.661)	(510.990)
Spread between Reichsbank and BoE official rates			− 26.927**	− 21.684*
			(13.396)	(11.421)
Trade Union Members Unemployed in UK, % (log)			− 93.210**	− 97.649**
			(39.440)	(39.183)
Constant	290.345***	282.069***	7404.490***	7878.006***
	(12.471)	(19.499)	(2090.060)	(2134.820)
Observations	300	300	300	300
Month indicators	No	No	No	No
Year indicators	Yes	Yes	Yes	Yes
Controls	No	No	Yes	Yes
*R*-squared		0.839		0.846
adjR2	0.825	0.825	0.830	0.829
*F* test model	873.2		492.5	
*P*-value of *F* model	0	0	0	0
First stage IV *F*-stat		103.1		69.89

The dependent variable is the total monthly credit authorised by the *Brasilianische* between 1889 and 1913 in thousand £. Standard errors are estimated with Newey–West and reported in parentheses. Column 1 shows the result of our OLS baseline model without additional control variables, while column 3 includes these variables. Columns 2 and 4 present the results of the respective IV regressions, without and with control variables

***p < 0.01, **p < 0.05, *p < 0.1

## Concluding remarks

This article studies the role of the LMM in determining the credit supply of non-British international banks in peripheral economies during the first wave of globalisation. At that time, London was the global financial hub, and sterling was the key international trade currency. Research and coeval studies have illustrated the importance of foreign banking in financing foreign trade of British competitors, such as Germany. At the same time, literature affirms that attempts to break the dominance of sterling and the LMM have failed. Yet, there seems to be a lack of quantitative research on the impact of the London Market dominance on the financial capabilities of non-British banks abroad.

Using the example of the German bank *Brasilianische Bank für Deutschland* in Brazil between 1889 and 1913, we find that the monthly credit lines authorised by the bank were positively related to the spread between the London market and the floating rate. Our findings suggest that the bank increases the provision of credit when there is either a rise in demand for foreign bills in the London market or a decrease in borrowing costs for bill brokers and discount houses in London. Even without a branch in London, the *Brasilianische* was able to benefit from the LMM thanks to its network of correspondents and agents, and, since 1901 from its mother institution, the *Disconto-Gesellschaft*.

We provide robust evidence that this relationship is causal. Firstly, we exclude possible issues of reverse causality since it is implausible that the *Brasilianische* had the capacity to influence conditions in the LMM. Secondly, the introduction of a large set of control variables that account for potential confounding effects does not change the results of our econometrical analysis. Thirdly, we develop an identification strategy (IV) based on the effect that the annual tax collection in Great Britain had on the liquidity conditions in the LMM and on the spread between the market and floating rate. Since the *Brasilianische* Bank was not subject to British taxes, we argue that this shock is exogenous.

These empirical results offer important new evidence and insights on the dynamics of the rising competition of economies challenging Great Britain’s global financial hegemony before WWI. It broadens our understanding of the development of financial centres, financial institutions, and their interdependence.

## Figures and Tables

**Fig. 6:**
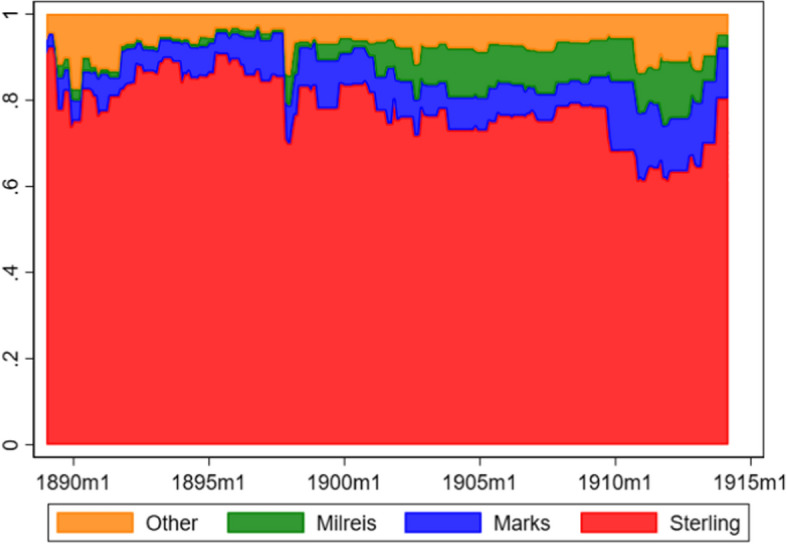
Breakdown by currencies of credit lines authorised by *Brasilianische* bank (1889–1913)

**Fig. 7:**
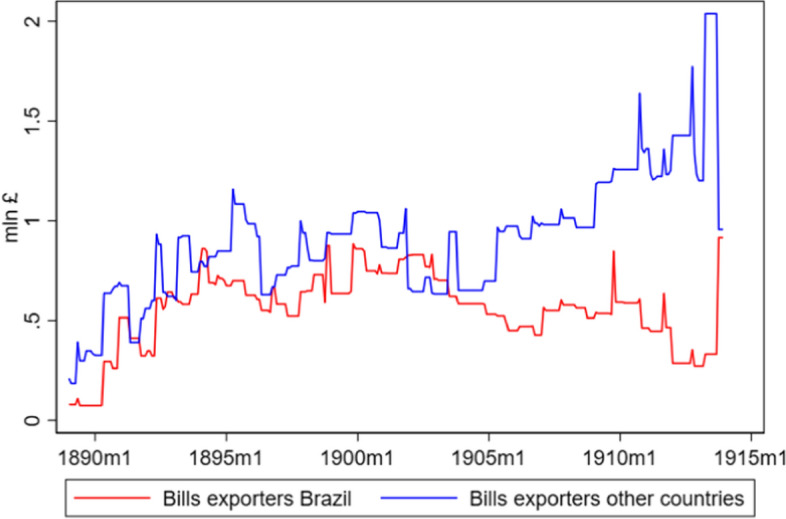
Authorised credit lines—bills discounted—by Brasilianische for exporters from Brazil and exporters from other countries—Monthly value in £ (1889–1913). All bills considered. Non £ bills converted to £ value. *Source*Kisling (2022)

## Appendix

See Appendix Figs. 6, 7, 8, 9, ^10^, 11 and Tables

**Table 9:** Bills receivable and discounted relative to deposits and credit current account of the Brasilianische Bank für Deutschland (DBRA) and the London & Brazilian Bank (LB)—monthly average ratio. *Source* own elaboration based on: The Brazilian Review, O Commercio de Sao Paolo, Jornal do Comercio Retrospecto, The Rio News, various years

	DBRA	LB
1893	0.63	0.8
1895–1900	0.99	0.95
1900–1905	1.17	0.56
1905–1910	1.79	0.94
1910–1913	2.27	1.69

^9^,

**Table 10:** Capital paid in (Milreis) Brasilianische Bank für Deutschland (DBRA) and the London & Brazilian Bank (LB). *Source* own elaboration based on: The Brazilian Review, O Commercio de Sao Paolo, Jornal do Comercio Retrospecto, The Rio News, various years

	DBRA	LB
1889–1895	1.115.00	5.556.00
1895–1910	10.000.00	13.333.00
1910–1912	10.000.00	17.777.00
1913	15.000.00	22.222.00

^10^,

**Table 11:** Deposit and credit current account (Milreis) Brasilianische Bank für Deutschland (DBRA) and the London & Brazilian Bank (LB)—monthly average ratio. *Source* own elaboration based on: The Brazilian Review, O Commercio de Sao Paolo, Jornal do Comercio Retrospecto, The Rio News, various years

	DBRA	LB
1893	11.841.97	14.663.09
1895–1900	24.844.03	23.208.32
1900–1905	15.321.79	23.219.61
1905–1910	18.056.80	18.427.04
1910–1913	29.796.48	23.506.95

^11^,

**Table 12:** Descriptive variables econometric analysis

Principal Variables	Obs	Mean	sd	min	max
Total credit in £, thousands	300	1,260.76	322.47	243	2,027.00
Spread between floating and discount rates, London	300	0.471	0.488	− 1.03	2.11
Bank of England public deposits (% change w.r.t. annual average)	300	0	31.53	− 48.99	107.535
*Main control variables*					
Mark/$ exchange rate	300	4.195	0.013	4.17	4.25
Spread between discount and call money rates, New York	300	1.43	1.197	− 1.45	6.75
Spread between Reichsbank and BoE official rates	300	− 0.842	0.629	− 2.4	1
Trade Union Members Unemployed in UK, % (log)	300	1.34	0.472	0.336	2.425
Additional control variables					
Official discount rate Reichsbank	300	4.255	0.994	3	7.5
Market discount rate in Berlin	300	3.31	1.137	1.26	7.07
Spread between German official and market discount rate	300	0.945	0.422	0.06	2.05
Spread between market discount rate in London and Berlin	300	− 0.535	0.803	− 2.68	1.52
Total Imports for Great Britain, mln £ (difference)	299	0.111	3.899	− 8.61	13.84
Total Exports of Produce and Manufactures for Great Britain, mln £ (difference)	299	0.076	2.178	− 7.8	7.4
Total exported bags of coffee from Rio de Janeiro (log) (difference)	299	0	0.349	− 1.25	0.995
Pig Iron Output for Germany, '000 metric tons (difference)	299	4.156	45.475	− 187.2	223.4
Clearings of Reichsbank for Germany, bln Marks (difference)	299	0.017	0.387	− 1.416	1.8
Earnings of Prussian-Hessian Railways from Freight for Germany, mln Marks (difference)	299	0.277	6.654	− 17.4	19.6
Value of Imports for Germany, mln Marks (difference)	191	2.791	53.935	− 196.7	217.33
Value of Exports for Germany, mln Marks (difference)	191	3.445	45.798	− 186	154
Security Price Index for London (difference)	299	0.045	0.813	− 4.5	4.3
Milreis/$ exchange rate (difference)	299	0.004	0.236	− 1.16	1.25
Total exported bags of coffee from Santos (log) (difference)	239	0.008	0.845	− 4.204	6.645
Exports, Total value for France mln Francs (difference)	287	1.39	58.47	− 239	154
Imports, Total value for France mln Francs (difference)	287	1.672	57.958	− 202	182
Total Exports for US mln $ (difference)	287	0.551	17.594	− 38.3	54.9
Total Imports for US mln $ (difference)	287	0.421	9.859	− 38.2	35.8

^12^,

**Table 13:** Augmented Dickey–Fuller test

Variables	Dfuller statistic	*p*-value	Dfuller statistic	*p*-value
			(1st difference)	(1st difference)
Total credit authorised in £, thousands	− 3.757	0.003		
Spread between floating and discount rates	− 2.853	0.051		
Bank of England public deposits (% change w.r.t. annual average)	− 7.069	0		
Spread between Reichsbank and BoE official rates	− 2.825	0.055		
Mark/$ exchange rate	− 3.041	0.031		
Spread between discount and call money rates, New York	− 4.871	0		
Trade Union Members Unemployed in UK, % (log)	− 2.961	0.039		
Total Imports for Great Britain, mln £	1.662	0.998	− 7.062	0
Total Exports of Produce and Manufactures for Great Britain, mln £	0.915	0.993	− 4.237	0.001
Total exported bags of coffee from Rio de Janeiro (log)	− 2.552	0.103	− 7.905	0
Pig Iron Output for Germany, '000 metric tons	0.991	0.994	− 4.014	0.001
Clearings of Reichsbank for Germany, bln Marks	3.485	1	− 4.544	0
Earnings of Prussian-Hessian Railways from Freight for Germany, mln Marks	2.888	1	− 6.035	0
Weights of Imports for Germany, '000 metric tons	− 0.320	0.923	− 4.038	0.001
Weights of Exports for Germany, '000 metric tons	1.815	0.998	− 4.174	0.001
Security Price Index for London	− 1.790	0.385	− 3.044	0.031
Milreis/$ exchange rate	− 1.568	0.5	− 4.679	0
Spread between German official and market discount rate	− 3.354	0.013		
Spread between market discount rate in London and Berlin	− 2.497	0.116		
Official discount rate Reichsbank	− 2.857	0.051		
Market discount rate in Berlin	− 3.950	0.002		
Price of coffee (log) $	− 2.033	0.272	− 3.610	0.006
Price of rubber (log) $	− 3.307	0.015	− 3.710	0.004
Total exported bags of coffee from Santos (log)	− 2.836	0.053	− 8.064	0.000
Exports, Total value for France mln Francs	0.776	0.991	− 4.952	0.000
Imports, Total value for France mln Francs	1.397	0.997	− 5.644	0.000
Total Exports for US mln $	2.541	0.999	− 1.699	0.432
Total Imports for US mln $	− 0.009	0.958	− 5.215	0.000

^13^,

**Table 14:** Results regression estimations—OLS—Eq. (1)—additional robustness checks

	(1)	(2)	(3)	(4)	(5)	(6)	(7)	(8)	(9)	(10)
	OLS with Newey–West S.E	OLS with Newey-West S.E	OLS with Newey-West S.E	OLS with Newey-West S.E	OLS with Newey-West S.E	OLS with Newey-West S.E	OLS with Newey-West S.E	OLS with Newey-West S.E	OLS with Newey-West S.E	OLS with Newey-West S.E
Spread between floating and discount rates	65.488***	56.682**	52.751***	50.796**	66.433***	58.558***	68.195***	58.428***	61.960***	54.530**
	(17.183)	(21.886)	(17.218)	(21.169)	(16.864)	(21.877)	(15.877)	(22.386)	(17.217)	(21.851)
Spread between Reichsbank and BoE official rates	− 24.331*	− 22.492*	− 24.714*	− 23.463*	− 23.303*	− 21.305	− 23.169*	− 21.297	− 21.759	− 22.090
	(13.938)	(13.443)	(13.729)	(13.810)	(13.533)	(13.227)	(13.564)	(13.217)	(18.174)	(17.340)
Trade Union Members Unemployed in UK, % (log)	− 98.006**	− 60.851	− 91.325**	− 52.406	− 94.110**	− 56.061	− 92.529**	− 56.698	− 126.581***	− 102.464**
	(42.028)	(46.133)	(41.492)	(50.542)	(42.296)	(48.267)	(40.578)	(47.559)	(45.401)	(48.151)
Exchange rate Mark/$	− 1750.779***	− 1506.162**	− 1705.836***	− 1495.201**	− 1782.792***	− 1507.638**	− 1739.729***	− 1497.610**	− 1745.045***	− 1793.522**
	(511.385)	(641.382)	(507.532)	(645.267)	(516.766)	(640.587)	(508.497)	(647.662)	(623.032)	(763.413)
Spread between discount and call money rates, NY	21.994**	21.595*	18.958**	21.161*	21.285**	20.284*	20.764**	20.244*	33.281*	29.204
	(10.167)	(11.996)	(9.254)	(11.721)	(9.946)	(11.346)	(10.097)	(11.376)	(19.295)	(21.741)
Total Imports for US mln $	0.617	1.226								
	(0.670)	(1.054)								
Total Exports for US mln $			0.858	1.254						
			(0.532)	(0.869)						
Imports, Total value for France mln Francs					0.103	0.043				
					(0.116)	(0.121)				
Exports, Total value for France mln Francs							0.127	0.025		
							(0.087)	(0.117)		
Total exported bags of coffee from Santos (log)									− 18.725*	− 19.367
									(9.607)	(12.509)
Price of coffee (log) $									432.407*	449.460*
									(236.660)	(248.125)
Constant	8069.047***	6959.002**	7876.870***	6928.080**	8199.419***	6962.581**	8016.398***	6921.250**	9046.451***	9174.459***
	(2139.571)	(2709.305)	(2121.661)	(2724.517)	(2163.318)	(2704.251)	(2128.495)	(2735.092)	(2,650.049)	(3,265.838)
Observations	287	287	287	287	287	287	287	287	240	240
Month indicators	No	Yes	No	Yes	No	Yes	No	Yes	Yes	Yes
Year indicators	Yes	Yes	Yes	Yes	Yes	Yes	Yes	Yes	Yes	Yes
Controls	Yes	Yes	Yes	Yes	Yes	Yes	Yes	Yes		
adjR2	0.729	0.725	0.731	0.726	0.729	0.723	0.729	0.723	0.607	0.595
*F* test model	232.3	161	205.9	157.8	229.2	158.8	220.9	159.4	274.8	155.9
*P*-value of *F* model	0	0	0	0	0	0	0	0	0	0

	(1)	(2)	(3)	(4)	(5)	(6)
	OLS with Newey–West S.E	OLS with Newey–West S.E	OLS with Newey–West S.E	OLS with Newey–West S.E	OLS with Newey–West S.E	OLS with Newey–West S.E
Spread between floating and discount rates—average between two meetings	129.303***	123.521***				
	(38.684)	(37.110)				
Spread between floating and discount rates—Assuming spread at time of last meeting prevails until next meeting			96.197***	93.475***		
			(31.854)	(35.228)		
Spread between Reichsbank and BoE official rates		− 25.225*		− 34.053**		− 35.273**
		(12.908)		(15.329)		(14.931)
Trade Union Members Unemployed in UK, % (log)		− 81.952**		− 76.219*		− 96.564**
		(41.212)		(42.912)		(44.625)
Exchange rate Mark/$		− 1,688.015***		− 1,739.460***		− 1,683.116***
		(536.607)		(605.033)		(595.493)
Spread between discount and call money rates, NY		15.209*		15.698		18.453*
		(9.052)		(11.467)		(10.473)
Average of the spread between *t*, *t*-1, and *t*-2					93.444***	92.516***
					(26.641)	(29.171)
Constant	267.859***	7,389.070***	282.726***	7,612.658***	283.517***	7,389.033***
	(17.150)	(2,241.283)	(14.152)	(2,529.729)	(14.232)	(2,488.810)
Observations	303	300	303	300	302	300
Month indicators	No	No	No	No	No	No
Year indicators	Yes	Yes	Yes	Yes	Yes	Yes
Controls	No	Yes	No	Yes	No	Yes
adjR2	0.834	0.836	0.831	0.835	0.824	0.829
*F* test model	2182	692.1	1417	388.3	5170	430.5
*P*-value of *F* model	0	0	0	0	0	0

Standard errors in parentheses, ****p* < 0.01, ***p* < 0.05, **p* < 0.1

^14^.

## Footnotes

1 It is important to note that joint-stock banks, when purchasing bills from the market, predominantly did so through bill brokers. This practice was a result of banks relying on these brokers to carry out the screening and assessment of the bill’s quality. We provide a more in-depth explanation of this process in Sect. 2 of our paper.

2 As stated by Neuburger and Stokes (1979): ‘In 1897 the largest exporting country was the United Kingdom (U.S.$1431.9 million) followed closely by the USA (U.S. $1153 million), Germany (U.S. $865.13 million) and then France (U.S. $694.41 million). […] The dominant position of the United Kingdom in exports at the beginning of the period was seriously eroded by the end. Although the United Kingdom still exported more than other countries, it appears that if the prevailing pattern continued, Germany would have overtaken the United Kingdom before long’. In Neuburger and Stokes (1979), ‘The Anglo-German Trade Rivalry, 1887-1913: A Counterfactual Outcome and Its Implications’, Social Science History, Vol. 3, No. 2. (Winter, 1979), pp. 187-201.

3 Dominated by the European industrial countries and their constantly increasing need for natural resources, agricultural products and new markets, Latin America exported primary products and raw materials in exchange for manufactured goods, among them military and industrial equipment (Bértola and Ocampo 2010, and Bulmer-Thomas 2003).

4 From the seventeenth century to well into the twentieth century, the Brazilian economy was heavily reliant on the production of primary commodities for export. Sugar was the dominant export product in the seventeenth and eighteenth century; and coffee and rubber were the most important leading export products from 1831 onwards (Hewlett 1975; Perkins & Weinstein 1985; Graham 1983; Absell & Tena-Junguito 2016). By 1913, its share in world coffee production reached 80 per cent ((Steven 2004; Krieger 2011). Brazil was the major global supplier of rubber for over a century, thanks to the abundance of the Hevea brasiliensis rubber tree in the Amazon rainforest. However, this dominance was increasingly challenged in the XX century, and Asian plantation exported more rubber than Brazil for the first time in 1913 (Resor 1977; Silva et al. 2011).

5 This work focuses on the role of foreign banking in Brazil during the first globalisation. Yet, the financial history of Brazil has been subject to various studies on different topics. Summerhill (2015) provides an in debt analysis of the (under-) development of Brazilian financial institutions and capital markets. Weller’s (2015) work focuses on the role of foreign institutions, namely Rothschild, in Brazil’s sovereign debt and government solvency. Musacchio (2009) looks at the development of capital markets from the perspective of investors, highlighting that despite weak laws, investors were relatively well protected in Brazil before 1940. Triner and Wandschneider (2005) study the contagion effect of the Baring Crisis in Argentina on Brazil in the early 1890s.

6 We have included a comparative analysis between the Rio de Janeiro branches of the Deutsche Brasilianische Bank and the British London & Brazilian Bank. Notably, by 1910, the Deutsche Brasilianische Bank had surpassed its British counterpart in terms of deposits and current accounts, despite starting with slightly less capital. This comparison is detailed in Tables 10 and 11 in the Appendix, highlighting the capital, deposits, and current account balances. For a more detailed comparison of the trade finance of British and German banks, see Kisling( 2017).

7 The supervisory board was responsible for final decision-making with regard to the bank’s operations. It was responsible for the appointment of staff, with the directorate in Brazil being in its entirety of German nationality (Brasilianische Bank für Deutschland 1912), and it controlled the bank’s strategy and provision of credit.

8 Including British Sterling, German Mark, French France, Italian Lire, and Brazilian Milreis. Figure 6 in the Appendix for a breakdown of major currencies over time. Figure 7 shows the authorised credit lines by the Brasilianische for exporters from Brazil and exporters from other countries.

9 BRA 1912, page 28. Translated from German by the authors.

10 There is indeed a vast literature on the role the Bank of England played in the LMM, repeating its role here would be redundant.

11 An old joke said ‘a pair of good boots and a bill case were the only capital that a bill broker had’. But this was in fact inaccurate, in fact, when bill brokers borrowed from London banks they normally deposited Treasury bills, Government bonds and other securities as collateral.

12 ‘Foreign Domicile’ were bills payable in London by a local bank but accepted in a different country than Britain. ‘Foreign Agency’ were bills accepted in London by branches of foreign banks not headquartered in Britain. The Bank of England did not accept this kind of paper at its discount window because in case the bills were dishonoured the assets that backed the signatures were in great part abroad.

13 The Economist (Oct. 20, 1900).

14 The Economist (Mar. 10, 1900).

15 Bankers’ Insurance Managers’ and Agents’ Magazine, 1903, September-October.

16 Available at the Historische Archiv der Deutschen Bank, Frankfurt am Main–Aktenzeichen KA/799—Brasilianische Bank für Deutschland—Sitzung des Aufsichtsrats—Sitzungssaal Norddeutsche Bank Hamburg.

17 The Brasilianische Bank für Deutschland opened its first branch in Rio de Janeiro in 1888, followed by branches in Sao Paulo (1893), Santos (1894), Porto Allegre (1904), and Bahia (1909). Information collected by authors from Lough (1915), and Brasilianische Bank für Deutschland (1912).

18 NBER Macrohistory Database: National Bureau of Economic Research, various variables, retrieved from FRED, Federal Reserve Bank of St. Louis.

19 As a robustness check, we also use the spread between the London market discount rate and the Berlin market discount rate. However, we prefer the specification with the spread between the official interest rate because to avoid mechanical correlation due to the inclusion of the same variable twice: The London market discount would be minuend in both spreads.

20 See: Maddison Project Database, Bolt and Zanden (2020).

21 We take this variable from Kisling (2020). As Santos increasingly became the main export port for coffee since the mid-1890s, also use ‘Total exported bags of coffee from Santos (log) (difference)’ instead of Rio de Janeiro. We hand-collected the data from the Journal do Commercio, but the data is available only from January 1984. Therefore, we put these estimates in Table 12.

22 From Global Financial Data Database: https://globalfinancialdata.com/ accessed on 25 October 2022.

23 The USA were the largest economy in terms of GDP throughout the entire period of investigation. Germany took over the United Kingdom, to become the second largest economy, in 1912 (see: Maddison Project Database, Bolt and Zanden 2020).

24 In NBER Macrohistory Database, these time series are available only from 1891. Ideally, we would have liked to use value instead of weights, but these statistics are not available at monthly level before 1909.

25 *‘Exports, Total value for France’, ‘Imports - Total Value for France’, ‘Total Exports for USA’* and ‘Total Exports for USA’, from the NBER Macrohistory Database.

26 One possible influence can be currency crisis such as that which occurred in the mid-1890s could have an impact on the Brazilian domestic economy, in turn affecting the demand for credit. In addition, Brazil issued bonds in London and therefore if depreciations in the currency triggered concerns about Brazil’s long-term solvency this might have repercussions in London too

27 The information for Milreis/$ exchange and price of rubber are from Global Financial Data Database: https://globalfinancialdata.com/ accessed on 25 October 2022.

28 Our IV approach also guarantees that the, if any, remaining concerns of revers causality are addressed.

29 Data on BoE public deposits are taken from Huang and Thomas (2016).

30 Banking and Currency, Sykes (1908).

31 See for example the ‘Money Market’ sections in The Economist, The Journal of the Institute of Bankers, or The Bankers’ Magazine in any issue around March to confirm this interpretation.

32 The first period run from January to the beginning of February, the second period from February until the beginning of April, the third period from April to September, the fourth and last period from September to December.

## References

[CR1] Abreu M, Bevilaqua A (1996) Brazil as an export economy, 1880–1930. In: Department of Economics, Catholic University of Rio de Janeiro

[CR2] Absell CD, Tena-Junguito A (2016) Brazilian export growth and divergence in the tropics during the nineteenth century. J Lat Am Stud 48(4):1–30

[CR3] Accominotti O, Lucena Piquero D, Ugolini S (2021) The origination and distribution of money market instruments: sterling bills of exchange during the first globalisation’. Econ Hist Rev 74(4):892–921

[CR4] Bértola L, Ocampo JA (2010) Desarrollo, Vaivenes y Desigualdad: una Historia Económica de América Latina desde la Independencia. SEGIB, Madrid

[CR02] Bignon V, Flandreau M, Ugolini S (2012) Bagehot for beginners: the making of lender-of-last-resort operations in the mid-nineteenth century 1. Econ Hist Rev 65(2):580–608

[CR5] Brasilianische Bank für Deutschland (1912) Brasilianische Bank für Deutschland. Hamburg—Brasilien. 1887–1912, Hamburg

[CR6] Briones I, Villela A (2006) European bank penetration during the first wave of globalisation: lessons from Brazil and Chile, 1878–1913. Eur Rev Econ Hist 10(3):329–359

[CR7] Bulmer-Thomas V (2003) The economic history of Latin America since independence. Cambridge University Press, Cambridge

[CR8] Carreras A, Josephson C (2010) Aggregate growth, 1870–1914: growing at the production frontier. In: Broadberry SN, O’Rourke K (eds) The Cambridge economic history of modern Europe, vol 2. Cambridge University Press, Cambridge, pp 30–58

[CR9] Clare G (1896) A money-market primer, and key to the exchanges with diagrams. E. Wilson, England

[CR10] Daudin G, Morys M, O’Rourke KH (2010) Globalisation, 1870–1914. In: Broadberry SN, O’Rourke K (eds) The Cambridge economic history of modern Europe, vol 2. Cambridge University Press, Cambridge, pp 5–29

[CR11] Dedinger B, Girard P (2017) Exploring trade globalization in the long run: The RICardo project. Hist Methods J Quant Interdiscip Hist 50(1):30–48

[CR12] Deutsche Überseeische Bank (1936) 50 Jahre Deutsche Überseeische Bank. Deutsche Überseeische Bank, Berlin

[CR13] Diouritch G (1909) L’Expansion de banques allemandes a l’etranger. Librarie Nouvelle de Droit et Jurisprudence, Paris

[CR14] Flandreau M, Jobst C (2005) The ties that divide: a network analysis of the international monetary system, 1890–1910. J Econ Hist 65(4):977–1007

[CR15] Graham R (1983) Comparing regional elites. A review article. Comp Stud Soc Hist 25:396–400

[CR16] Haber SH (1997) Financial markets and industrial development. A comparative study of governmental regulation financial innovation and industrial structure in Brazil and Mexico 1940–1930. In: Haber SH (ed) How Latin America fell behind: essays on the economic histories of Brazil and Mexico: 1800–1914 (pp. 146–178). Stanford University Press

[CR17] Hauser R (1901) Die Deutschen Überseebanken. In: Pierstorff J (ed) Abhandlung des Staatswissenschaftlichen Seminars zu Jena. Verlag Gustav Fischer, Jena

[CR18] Hertner P (2012) German banks abroad before 1914. In: Jones G (ed) Banks as multinationals. Taylor & Francis Group, London

[CR19] Hewlett S (1975) The dynamics of economic imperialism: the role of foreign direct investment in Brazil. Lat Am Perspect 2:136–148

[CR20] Huang H, Thomas R (2016) The weekly balance sheet of the Bank of England 1844–2006, OBRA dataset, Bank of England

[CR21] Hurley EN (1914) Banking and Credit in Argentina, Brazil, Chile and Peru (Special Agents Series No. 90). U.S Department of Commerce, Washington D.C

[CR22] Jones G (1993) British multinational banking 1830–1990. Clarendon Press, Oxford

[CR23] Kindelberger C (1974) The formation of financial centers: a study of comparative economic history. In: Princeton studies in international finance, 36, Princeton University

[CR24] King W (1972) History of the london discount market. Cass, London

[CR25] Kisling W (2020) A microanalysis of trade finance: German bank entry and coffee exports in Brazil, 1880–1913. Eur Rev Econ Hist 24(2):356–389

[CR26] Kisling W (2023) Los Von London”: a comparative, empirical analysis of German and British global foreign banking and trade development, 1881–1913. Econ Hist Rev 76(2):445–476

[CR27] Kisling W (2017) La financiación del comercio: bancos alemanes y británicos en el Brasil del siglo XIX. In: Fuentes DD, Aparicio AH, Marichal C (eds.) Orígenes de la Globalización Bancaria Experiencias de España y América Latina, pp. 179-204, El Colegio de México, Genueve Ediciones

[CR28] Klasing MJ, Milionis P (2014) Quantifying the evolution of world trade, 1870–1949. J Int Econ Elsevier 92(1):185–197

[CR29] Krieger M (2011) Kaffee: Geschichte eines Genussmittels. Böhlau, Wien

[CR30] Lough WH (1915) Banking Opportunities in South America. Department of Commerce, Bureau of Foreign and Domestic Commerce, Special Agents Series No. 106

[CR31] Musacchio FA (2009) Experiments in financial democracy : corporate governance and financial development in Brazil, 1882–1950. Cambridge University Press

[CR32] Neuburger H, Stokes HH (1979) The Anglo-German trade rivalry 1887–1913: a counterfactual outcome and its implications. Soc Sci Hist 3(2):187–201

[CR33] Nishimura S (1971) The decline of inland bills of exchange in the London money market, 1855–1913. Cambridge University Press, London

[CR01] Orbell J, Turton A (2001) British banking: a guide to historical records. Studies in British business archives. Ashgate, Aldershot

[CR34] Peake E (1926) An academic study of some money market and other statistics, 2nd edn. P.S. King, London

[CR35] Perkins P, Weinstein B (1985) The Amazon rubber boom 1850–1920. Technol Cult 26:865

[CR36] Resor R (1977) Rubber in Brazil: dominance and collapse, 1876–1945. Bus Hist Rev 51:341–366

[CR37] Schneider S (2019) Imperial Germany, Great Britain and the political economy of the gold standard, 1867–1914. In: Hoppit J, Needham D, Leonard A (eds) Money and markets: essays in honour of Martin Daunton. Boydell & Brewer, pp 127–144

[CR38] Silva C, Campos T, Scaloppi E, Maluf M, Gonçalves P, Souza A (2011) Construction and analysis of a leaf cDNA library from cold stressed rubber tree clones. BMC Proc 5:P24–P2422373222

[CR39] Spalding W (1922) The London money market: a practical guide to what it is, where it is, and the operations conducted in it. Sir I. Pitman & Sons, London

[CR40] Strasser K (1924) Die Deutschen Banken im Ausland, PhD Dissertation, University of Lausanne

[CR41] Summerhill WR (2015) Inglorious revolution. Yale University Press, New Haven

[CR42] Sykes E (1908) Banking and currency, 2nd edn. Butterworth, London

[CR43] Tilly R (1992) An overview on the role of large German banks up to 1914. In: Cassis Y (ed) Finance and financiers in European history 1880–1960. Cambridge University Press, pp 93–112

[CR44] Steven T (2004) The world coffee market in the eighteenth and nineteenth centuries, from colonial to national regimes, working paper no. 04/04, LSE, Department of Economic History

[CR45] Triner GD, Wandschneider K (2005) The baring crisis and the Brazilian encilhamento, 1889–1891: an early example of contagion among emerging capital markets. Financ Hist Rev 12(2):199–225

[CR46] Triner GD (2006) British Banks in Brazil during an Early Era of Globalisation (1889–1930). In: Prepared for: European banks in Latin America during the first age of globalization, 1870–1914, session 102, XIV international economic history congress Helsinki

[CR47] Bolt J, van Zanden JL (2020). Maddison style estimates of the evolution of the world economy. A new 2020 update In: Maddison project database, version 2020

[CR48] Weller L (2015) Rothschilds’ “delicate and difficult task”: reputation, political instability, and the Brazilian rescue loans of the 1890s. Enterp Soc 16(2):381–412

[CR49] Young GF (1991) British Overseas banking in Latin America and the encroachment of German competition, 1887–1914. Albion Q J Concerned Br Stud 23(1):75–99

